# Recent applications of mass spectrometry in the analysis of transformation products of emerging contaminants in PM_2.5_

**DOI:** 10.1002/ansa.202200038

**Published:** 2023-02-08

**Authors:** Wei Wang, Guodong Cao, Jing Zhang, Han Qiao, Fuyue Wang, Zongwei Cai

**Affiliations:** ^1^ State Key Laboratory of Environmental and Biological Analysis Department of Chemistry Hong Kong Baptist University Hong Kong SAR China

**Keywords:** air pollution, emerging contaminants, fine particulate matter (PM_2.5_), mass spectrometry, transformation products

## Abstract

Ambient pollution correlated to fine particulate matter (PM_2.5_) is a worldwide environmental issue as it is highly associated with human health and eco‐environmental safety. A significant part regarding the toxicity of PM_2.5_ is attributed to its bonded contaminants. Appreciable efforts have been performed to study the occurrence, exposure, and toxicological properties of chemicals of emerging concerns in PM_2.5_. Recent works indicated a broad environmental transformation of emerging contaminants in the atmospheric environment and highlighted the significance of PM_2.5_ bonded transformation products, which may exhibit higher environmental concentrations and toxicities compared to their parent compounds. Among these studies, mass spectrometry has been widely applied for the analysis of transformation products of emerging contaminants in PM_2.5_ on the aspects of suspect/non‐target screening, structure elucidation, concentration profiling, and toxicity determination. This review describes key mass spectrometry‐based analytical strategies and applications for determining transformation products in PM_2.5_ and presents outlooks for their analysis.

## 1 INTRODUCTION

Atmospheric pollution associated with fine particulate matter (aerodynamic diameter less than 2.5 µm, PM_2.5_) has caused serious concerns globally during the last century, as several adverse outcomes including malignancies, pulmonary, cardiovascular, and central nervous system disorders, are highly correlated with it.^1^, ^2^, ^3^ Given these facts, ambient contamination with high levels of PM_2.5_ is considered one of the five most significant health risks worldwide.^4^ Due to its large specific surface area, PM_2.5_ can absorb a wide range of organic molecules while being a complex matrix that is significantly affected by its environment.^5^, ^6^ As a consequence, quite a part of the PM_2.5_‐induced toxicity is proven to be associated with multiple bonded contaminants, for example, polycyclic aromatic hydrocarbons (PAHs), organohalogen compounds, organophosphate esters (OPEs), chlorinated paraffins (CPs), and substituted *para*‐phenylenediamines.^7^, ^8^ To mitigate the potential risks caused by these chemicals in the surrounding atmosphere, people have listed chemicals of emerging concerns to prioritize and further control them. Many efforts have been made to monitor the environmental levels, estimate the exposures and hazard quotients, and investigate the toxicological consequences of these emerging contaminants. As research continues to progress, the transformation behaviors of a variety of emerging contaminants in the atmospheric environment have been increasingly realized, including hydrolysis, photolysis, oxidation, and biodegradation, revealing prevalent occurrences of numerous transformation products (TPs) bonded to PM_2.5_. Due to the intensive irradiation intensity and strong oxidation capacity, atmosphere environment could highly promote the photoreaction and ozonation, making the TPs have high‐complicated variety, composition and physicochemical properties.^9^, ^10^ Thus, discovering and characterizing the transformation products bonded to PM_2.5_ is of great significance and become an increasingly popular spot in the field of environmental science.

Mass spectrometry (MS), as a powerful analytical tool, has been widely applied in determining novel transformed pollutants in PM_2.5_.^11^, ^12^, ^13^, ^14^, ^15^ Different from the traditional techniques such as thin‐layer chromatography and gas/liquid chromatography (GC/LC) with unspecific detectors, MS techniques with high specificity can identify and elucidate the TPs with accurate molecular masses of both precursor compounds and fragments. By interfacing with multiple ionization techniques, especially soft ionization sources including matrix‐assisted laser desorption ionization (MALDI), (atmospheric pressure) chemical ionization (APCI/CI), and electrospray ionization (ESI), high‐resolution mass spectrometry (HRMS) could deconvolute the sample with thousands of specific molecular information with high confidence analyte identification from a single analysis. MS could also be used to yield characteristic fragments of target analytes by combining with a variety of fragmentation techniques such as collision‐induced dissociation (CID), higher‐energy collisional dissociation (HCD), and electron transfer dissociation (ETD), which promotes the structural elucidation of the unknown emerging TPs. Existing evidence indicated that different dissociation methods would induce different ion types, thus an integrated approach can provide complementary information for strengthening analyte identification and structural characterization.^16^ Additionally, triple quadrupole (QqQ) MS with high sensitivity and selectivity enables the quantification of trace level TPs among different environmental matrices, which helps to build their environmental concentration and compositional profiles. Last but not least, studies on the environmental contamination‐related health effects also highly relied on the advanced MS instruments, in the aspects of metabolites monitoring, adducts identification, and biomolecules spatial distribution analysis.^17^, ^18^, ^19^

Hereby, we give an overview of the recent 5‐year (2017‐2022) applications of MS in the analysis of emerging transformation contaminants in PM_2.5_ by (1) outlining the MS‐based analytical strategies for the discovery of transformation products in PM_2.5,_ (2) summarizing recent progress in the analysis of PM_2.5_‐bonded transformation products using MS and (3) prospecting for the future applications of MS in characterizing the emerging transformation products in PM_2.5_.

## 2 MASS SPECTROMETRY‐BASED STRATEGIES FOR THE DISCOVERY OF TRANSFORMATION PRODUCTS IN PM_2.5_

**FIGURE 1:**
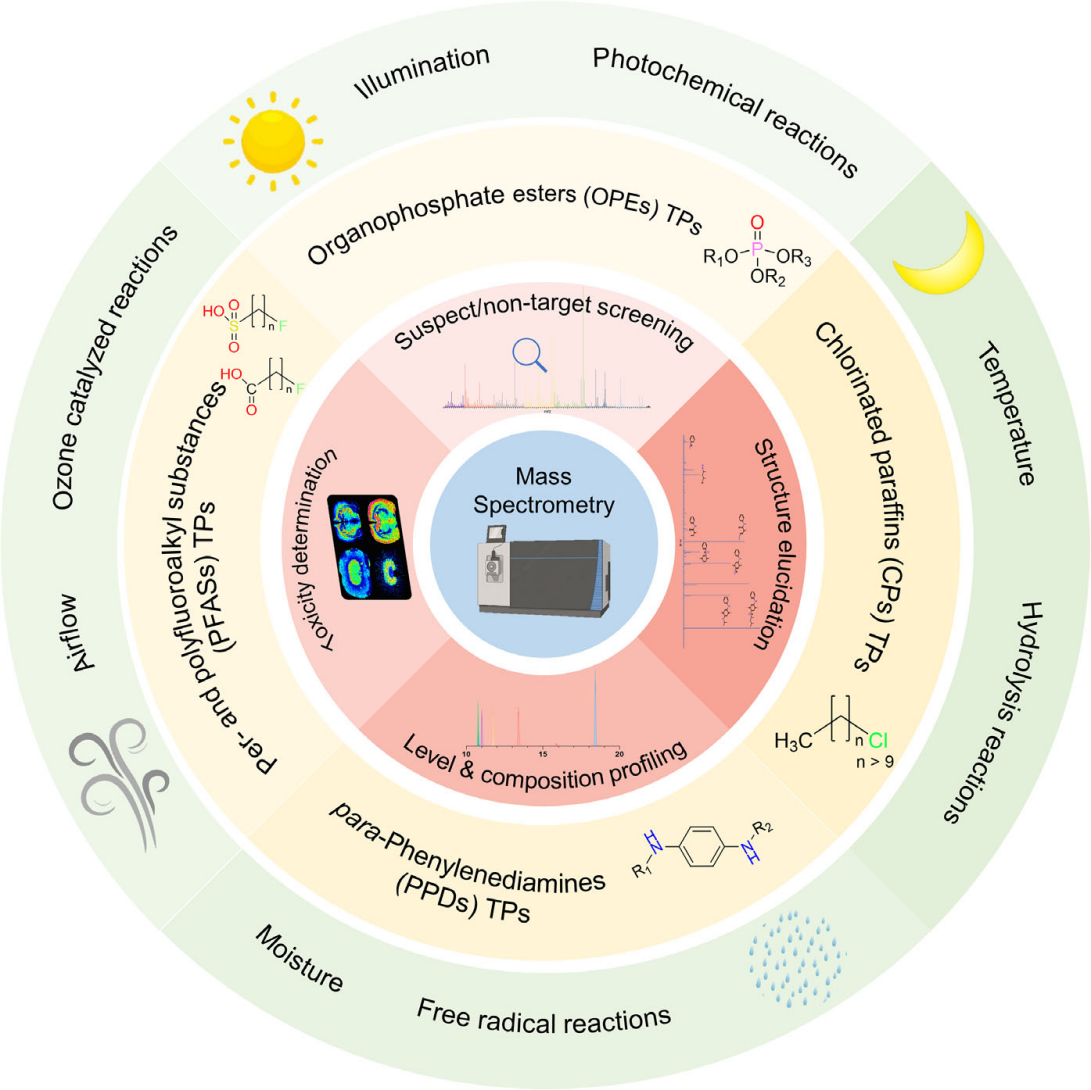
Mass spectrometry‐based strategies in the analysis of diverse transformation products of emerging contaminants in PM_2.5._ Different MS strategies (inner circle in red) are applied to investigate the transformation of multiple emerging contaminants (middle circle in yellow) via various atmospheric reactions (outer circle in green).

Analytical strategies of PM_2.5_‐bonded transformation products with MS platforms could be summarized in four aspects: (1) suspect/non‐target screening; (2) structure elucidation; (3) concentration profiling and (4) toxicity determination. As a powerful tool in contamination screening, MS was widely used in the field of atmospheric environmental chemistry as Figure 1 shows. Suspect and non‐target screening are the major methods to identify novel pollutants that were previously unattainable. By applying high‐resolution mass analyzers (e.g., time‐of‐flight [TOF], orbitrap, and Fourier transform‐ion cyclotron resonance [FT‐ICR]) with high mass accuracy, selectivity, and range, massive molecular information, for example, accurate mass, and elemental composition, could be obtained through a single data‐dependent/‐independent acquisition (DDA/DIA) analysis.^20^, ^21^ These acquisition modes enable a high‐quality identification of suspect/unknown transformation products, without requiring reference standards available or even without setting the preselection of analytes.^20^ In addition, a high performance chromatographic separation before MS analysis is crucial during this process, as highly complex atmospheric samples would be better characterized by reducing co‐eluting species. The ionization suppression would be reduced thus yielding higher detection sensitivity, lower matrix effect, and wider dynamic range of the measurement. Besides screening and identifying the molecular features of atmospheric transformation chemicals, HRMS are also used in determining the chemical structures of emerging TPs through the characterization of their unique product fragments.^12^ By coupling with quadrupole to add MS^2^/MS^n^ capabilities, mass spectra of product ions with accurate masses and abundant functional group information could be attained by HRMS measurements, particularly using parallel reaction monitoring (PRM) acquisition.^20^ Compared to DDA/DIA analysis, PRM allows richer fragmentation information accompanied by changing collision energies and reducing matrix effects, which would yield higher sensitivity and highly promote the structure elucidation of the unknowns.^22^ In contrast to DDMS,^2^ where precursor ions are isolated in the quadrupole to generate product ions in the collision cell only when a signal threshold has been reached, PRM data acquisition continuously isolates precursor ions for all target compounds regardless of their intensity, which enabled a high sensitive and informative confirmation of identity.^23^ An adequate chromatographic separation is also significant in this strategy, as it ensures effective ion acquisition and provides abundant fragmentation data that highly promote the elucidation of structures.^24^ Besides qualification, MS could also be used as a quantitative mean. By applying the obtained precursor‐product information of targeted TPs as selection criteria, the abundance of target compounds could be attained in a single MS/MS run with the exclusion of co‐eluted matrix compounds or noisy peaks. Several technologies, including background subtraction, dynamic exclusion, and charge state selection, could be applied in this procedure to avoid obtaining the product ion spectra of the interferences or the redundant collection of the most abundant ions.^25^ Many studies have investigated the concentration levels and compositional profiles of the environmental TPs, by applying this tandem MS approach with multiple/selective reaction monitoring (MRM/SRM) scan mode.^26^ Besides that, MS has also been applied in the characterization of toxicities and its mechanisms of atmospheric TPs, by screening in vivo metabolites and adducts, to further investigate their bio‐accumulations and distributions.^17^, ^18^ For these multiple reasons, MS is regarded as a potent technique that extends the horizon in screening, identification, quantification and determination of a wide range of unknown atmospheric transformation products.

## 3 RECENT PROGRESS IN THE ANALYSIS OF PM_2.5_‐BONDED TRANSFORMATION PRODUCTS USING MASS SPECTROMETRY

Recent investigations have revealed the transformation behavior of a variety of PM_2.5_ bonded emerging contaminants, including OPEs, CPs, per‐ and polyfluoroalkyl substances (PFAS), and *para*‐phenylenediamine antioxidants (PPDs). By utilizing diverse MS‐based analytical strategies, the transformation mechanisms and relevant products of these emerging contaminants were specifically characterized.

### 3.1 Transformation products of OPEs

OPEs represent a series of organophosphorus compounds and are broadly applied as flame retardants, plasticizers, and performance additives.^27^, ^28^ As good substitutes for polybrominated diphenyl ethers (PBDEs), OPEs are largely consumed (over 6800000 tons cumulative global consumption by 2015),^29^ and can release from products to the environment.^30^ By using GC‐MS/MS operated at MRM mode, the level of OPEs in the atmospheric environment (e.g., indoor air and atmospheric particulates) were quantified and the results revealed a ubiquitous occurrence of that among different countries such as Sweden (3.1 × 10^3^ pg·m^−3^), Canada (1.8 × 10^3^ pg·m^−3^), Turkey (4.4 × 10^3^ pg·m^−3^), China (1.6 × 10^5^ pg·m^−3^) and the U.S. (1.4 × 10^3^ pg·m^−3^).^31^, ^32^, ^33^, ^34^ Given the frequent occurrence of OPEs in PM_2.5_, as well as the high reactivity of the atmospheric environment, the transformation of OPEs in PM_2.5_ are widely identified using MS‐based approaches.^35^, ^36^ Recently, By using UPLC ESI QqQ MS in MRM mode, Liu et al., have quantified a series of OPEs in indoor dust collected in Canada, and confirmed the transformation route from tris(2,4‐di‐tert‐butylphenyl) phosphite (AO168) to tris(2,4‐di‐tert‐butylphenyl) phosphate (AO168 = O), and further hydrolysis to bis(2,4‐di‐tert‐butylphenyl) phosphate (B2,4DtBPP) and 2,4‐di‐tert‐butylphenol (2,4DtBP).^36^ These transformation products were further founded in multiple environmental compartments including indoor/outdoor dust, airborne particles, and soils, by using HPLC‐ESI QqQ MS.^15^, ^37^ Notably, quantification results obtained from UPLC‐ESI MS/MS using MRM mode indicated a surpassed level of 2,4DtBP (113000 ng/g) in airborne particles than its precursor AO168 = O (52900 ng/g), implying an exceed human exposure through inhalation.^35^ Similarly, Lao et al. have utilized the LC quadrupole TOF (qTOF) MS operated at DIA mode to suspect screening of the TPs of PM_2.5_‐bonded OPEs in Hong Kong.^13^ The scanning pattern with dynamic background subtraction enables full scan and simultaneously triggers MS^2^ data acquisition. The result revealed a large array of oxidation products of OPEs and proposed potential atmospheric ozonation mechanisms. Besides that, by using a high‐sensitive LC‐MS/MS approach, a considerable level (1520‐3740 pg·m^−3^) of OPE‐derived transformation products was determined. A specific compositional profile of TPs with different substitutions was demonstrated in which alkyl‐OPEs showed a high occurrence rate and abundance (<reporting limits‐3540 pg·m^−3^), while Cl‐OPEs and aryl‐OPEs were lower than the reporting limits. These observations were rationalized by the rapid microorganism degradation and/or easily •OH oxidation of Cl‐OPEs.^38^

**FIGURE 2:**
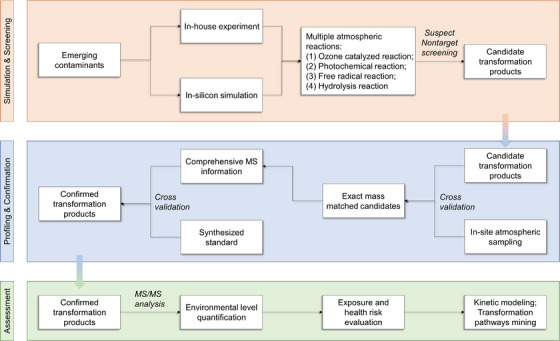
Mass spectrometry‐based framework for identifying the potential transformation products and assessing their environmental risks of atmospheric emerging contaminants. Level 1: Multiple atmospheric reactions are considered in the in‐house experiment and in‐silicon simulation, followed by suspect/nontarget MS screening; Level 2: The candidate products are validated with in‐site collected samples and confirmed with synthesized standards using comprehensive MS information (e.g., MS^2^ ions, fragmentation patterns); Level 3: The identified transformation products would be determined in their environmental levels for health risk evaluation and kinetic modeling.

Besides the traditional identification‐quantification pattern of TPs determination, a more effective framework combining in‐house experiments, advanced MS screening techniques, and in‐silicon modeling was recently built.^14^ This approach would effectively promote the prediction of airborne TPs and the evaluation of their exposure risks with full consideration of multiple atmospheric chemical reactions (summarized as Figure 2). By coupling this framework with gas chromatography atmospheric pressure chemical ionization (GC‐APCI) qTOF MS‐based non‐target analysis (NTA) of atmospheric OPEs, a total of 186 TPs were identified, which were proven to be globally distributed across 18 megacities. As a soft ionization technique, APCI can maintain the analyte in the pattern of [M]^+^ or [M+H]^+^ via charge exchange between N_2_^+^ and analyte molecules M or proton exchange between H_3_O^+^ and M, which enables the identification of molecular ions with fewer fragments compared to the traditional GC‐EI MS. This result not only uncovered the unrecognized exposure risk for urban populations globally but also revealed the possibility of individual TPs being more hazardous and persistent than their parent chemicals, leading to a higher level of combined risks compared to their parent compounds.

### 3.2 Transformation products of CPs

CPs are a class of emerging organic pollutants with a chlorination degree of 30‐70%, and can be categorized according to their carbon chain length into short‐chain CPs (SCCPs, C_10_‐C_13_), medium‐chain CPs (MCCPs, C_14_‐C_17_), and long‐chain CPs (LCCP, C_18_‐C_30_).^39^ Among them, SCCPs have been particularly recognized as novel persistent organic pollutants (POPs) by the Stockholm Convention since 2017, considering their persistence, high toxicity, and bio‐accumulative potential.^40^ CPs are considered to be persistent at ambient temperature but subject to attack via indirect photolysis by oxidizing radicals in the troposphere.^41^

**FIGURE 3:**
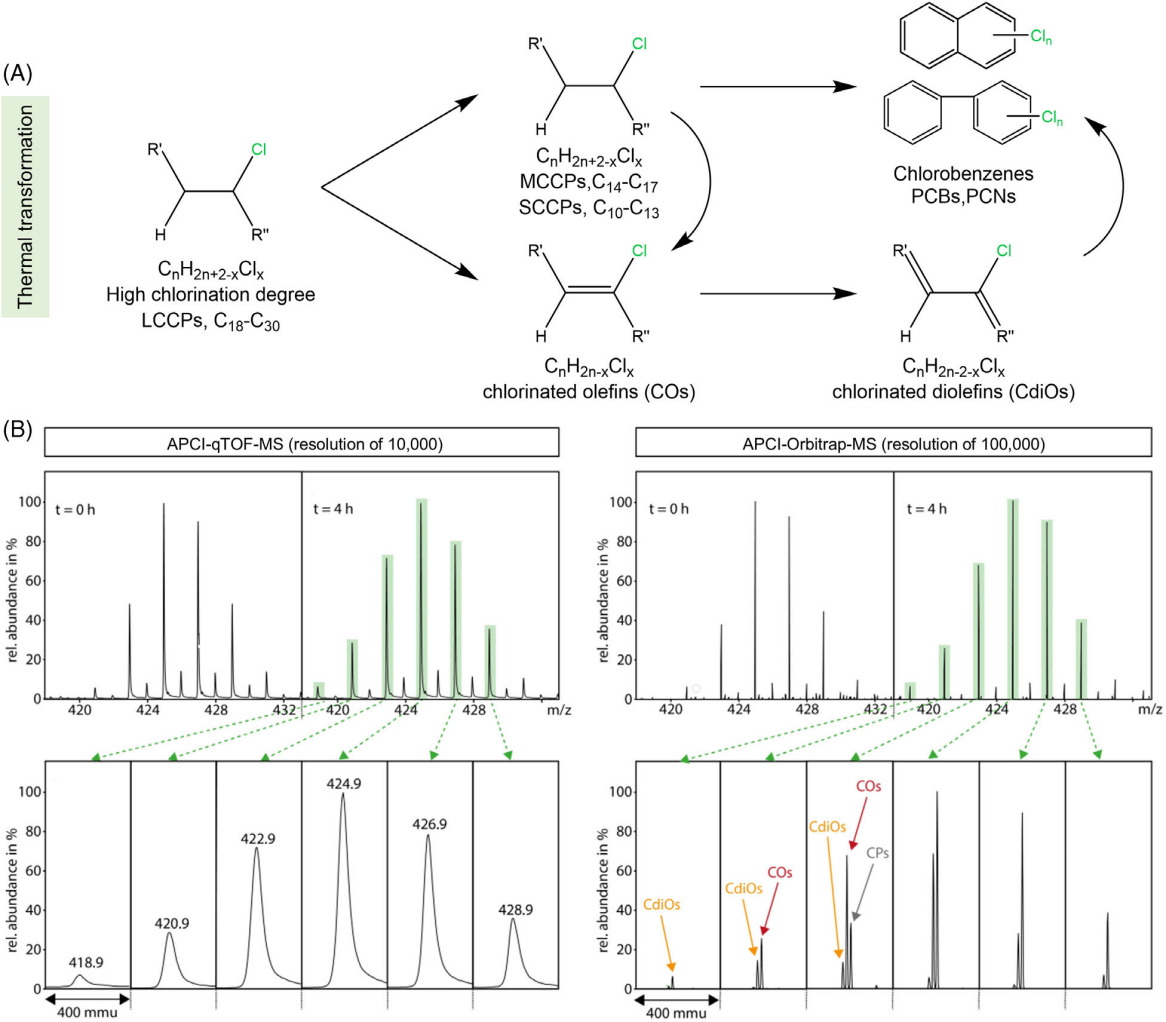
(A) Thermal transformation of CPs in the environment. (B) Comparison of isotope clusters of CPs (before and after thermal exposure), COs and CdiOs measured with APCI‐qTOF‐MS and APCI‐Orbitrap‐MS. Zooms (400 mmu) to single isotope signals show that Orbitrap‐MS with a resolution of 100000 can resolve these mass interferences whereas the used qTOF‐MS with a resolution of 10000 cannot. Reprint with permission from^46^

It was early investigated the indirectly‐photolysis of CPs using solid phase extraction (SPE) followed by GC‐EI quadrupole MS, which indicated the degradation of long‐chain CPs to short‐chain CPs, and furtherly transformed into low molecular products such as polychlorinated biphenyls and naphthalene under thermal stress.^42^ Recently, by combing the fragment‐abundant EI source and molecular ion‐abundant CI source with high‐resolution GC TOF MS (Resolution = 15000 at *m/z* 300‐600), the decomposition of a highly CP_70_ was studied.^43^ With the exact mass obtained from HRMS, several TPs were identified and further semi‐quantified such as SCCPs, MCCPs, unsaturated analogs (Cl‐polyenes), and toxic chlorinated aromatic hydrocarbons (Figure 3A). Among these TPs, chlorinated polyenes especially chlorinated olefins (COs) and chlorinated di‐olefins (CdiOs) are identified as the thermal transformation products of CPs found in CPs‐relevant products and surrounding environment. These compounds would mislead the quantification of CPs due to the in‐source degradation of CPs and very close molecular weights between CPs and COs/CdiOs when using conventional gas chromatography electron capture negative ionization mass spectrometer (GC‐ECNI‐MS).^44^, ^45^ Figure 3B demonstrated the full scan of thermolysis of chlorinated tridecane mixtures samples analyzed with qTOF‐MS (resolution of about 10000) and Orbitrap‐MS (resolution of about 100000).^46^ It is clear that Orbitrap‐MS with higher resolution can resolve mass interferences caused by thermolysis products of CPs including COs and CdiOs, while qTOF‐MS cannot. Several subsequent developed mathematical processes enable the deconvolution of equipment unsolved ion clusters into linear combined groups with respective CP, CO, and CdiO compounds and have obtained similar compositional profile information.^44^, ^45^ To address the in‐source degradation and insufficient resolution problems, alternative methods based on LC and soft ionization sources, coupled with high‐resolution MS were established and these investigations clarified a board occurrence of COs/CdiOs in non‐exposed CP products, as well as atmospheric particulate samples.^47^ Li et al. have utilized UPLC‐APCI/ESI technology followed by Orbitrap MS (resolution of about 140000) to characterize the detailed compositions of CPs commercial mixtures.^48^ Chromatographic retention time, peak shape, and isotope ratio were adopted as criteria to identify congener groups of CPs. Combined with the characteristic isotope chlorine peaks of CPs, several saturated/unsaturated CPs were discovered in air samples and their concentrations were (semi‐) quantified.

### 3.3 Transformation products of per‐ and polyfluoroalkyl substances (PFAS)

Per‐ and polyfluoroalkyl substances (PFAS, C_n_F_2n+1_‐R) are a broad category of chemicals with multiple applications in manufacture and commerce,^49^ and are listed as POPs due to their persistence, toxicity, bioaccumulation, and potential for long‐range transport.^50^ Consequently, traditional PFAS were restricted in use and the production/consumption has shifted toward novel PFAS (e.g., chlorinated polyfluorinated ether sulfonates, Cl‐PFESAs),^51^ raising new concerns about their environmental levels and potential toxicities.^52^

**FIGURE 4:**
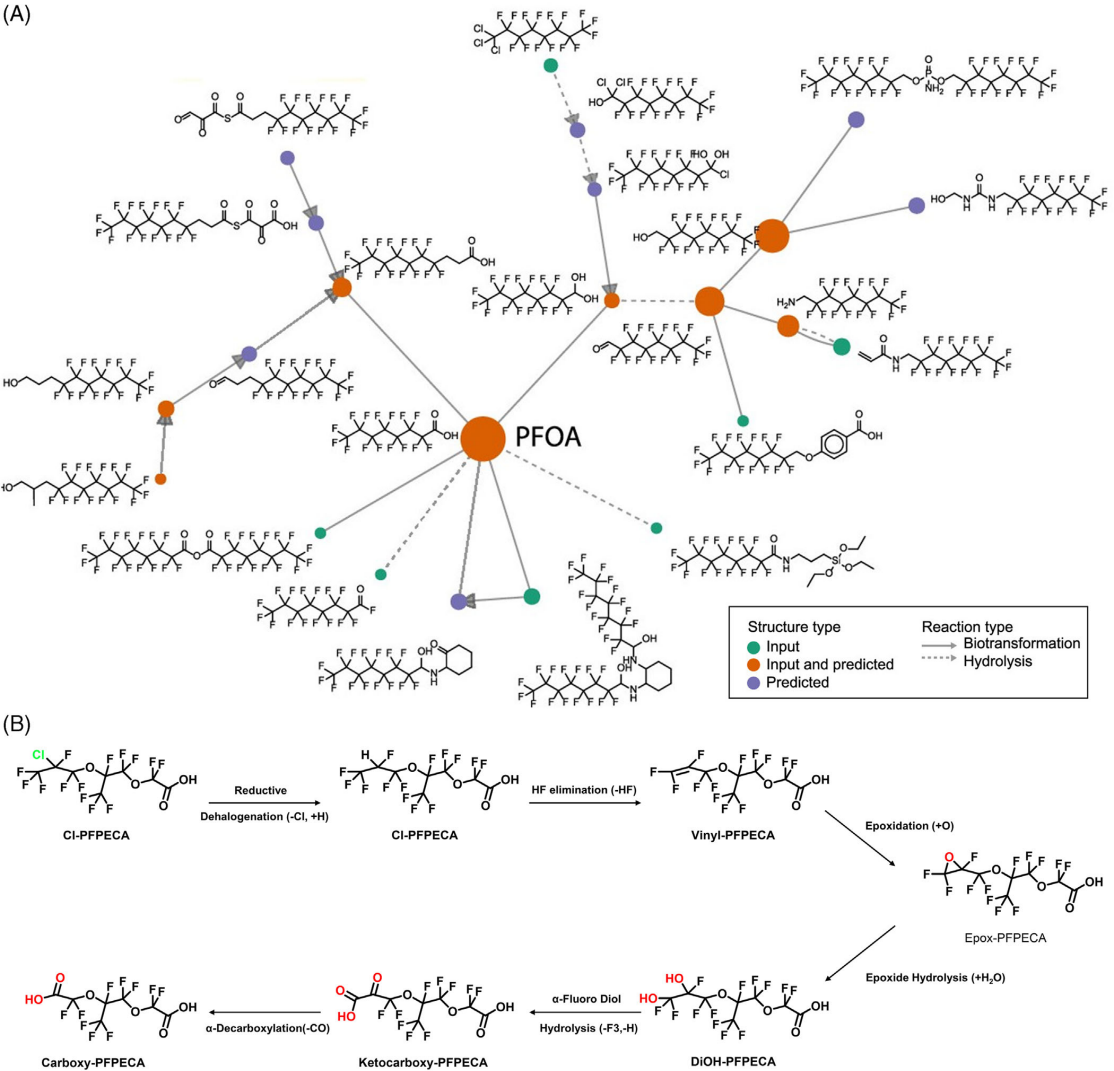
(A) Molecular network of PFOA in the predicted reactions. Nodes represent unique chemical structures, in which green nodes represent structures from PFAS molecular databases, purple nodes represent molecules predicted by transformations, orange nodes represent structures present in input molecular databases and predicted by in silico transformations. The predicted parent–product relationships from either biotransformation (solid arrows) or hydrolysis (dashed arrows) are indicated with different arrows. (B) Predicted transformation pathway of the Cl‐PFPECA congener containing a single propyl unit. Reprint with permission from^56^

PFAS share similar elemental compositions with perfluoroalkyl moieties, which makes them particularly persistent in the environment.^53^ Therefore, most PFAS are either not degradable or transformed into stable terminal TPs.^54^ As former studies have summarized well about the bio‐ and environmental transformation of PFASs, here we only covered very recent progress in MS‐based identification of PFAS’ transformation products.^19^, ^55^ Similar to OPEs, recent studies also established a database containing the molecular network of PFAS (specifically with perfluorooctanoic acid, PFOA), and their biotic/abiotic TPs with MS fragmentation spectral library predicted through in‐silico calculations (Figure 4A).^56^ This work demonstrated the efficacy of the combined platform with HRMS‐based suspect‐ and non‐target screening and machine learning‐based TPs prediction. Similarly, a very recent study developed a reaction library‐based generalized reaction scheme to anticipate the probable TPs and metabolites of PFAS described in the peer‐reviewed literature in a variety of environmental and biological reaction systems.^57^ Besides that, based on the newly identified PFAS alternative, chloroperfluoropolyether carboxylates (Cl‐PFPECAs) reported by Washington et al. in *Science*,^58^ a further investigation using qTOF HR MS‐based non‐targeted screening and literature reports‐based TPs prediction was conducted.^59^ According to the generated transformative structures and screening results obtained from the NTA of HR LC‐MS/MS, a series of Cl‐PFPECAs TPs and their congeners with different functional groups, that is, hydroxy‐, vinyl‐, expo‐, and dihydroxy‐ substituted Cl‐PFPECAs were tentatively observed as the proposed PFAS transformation schemes demonstrated (Figure 4B). The concentration of Cl‐PFPECAs and identified TPs was further semi‐quantified by normalizing their chromatography peak area ratios compared to the internal matrix standard (mass‐labeled perfluorononanoic acid, ^13^C_5_‐PFNA). It is proposed that a chlorine‐to‐hydrogen substitution during reductive dehalogenation was a plausible transformation mechanism based on the observed similarities between these found congeners and Cl‐PFESAs.

### 3.4 Transformation products of substituted *para*‐Phenylenediamines (PPDs)

Substituted *para*‐phenylenediamines (PPDs) are a class of anthropogenic antioxidants, which are widely utilized in rubber industries given their superior performance in protecting rubber products.^60^ The wide appliance of PPDs has spawned its massive production and consumption, many of which were listed as high‐production volume chemicals.^61^ Nevertheless, massive production/consumption of PPDs has caused increasing concerns regarding their environmental occurrences and ecological risks, with emerging evidence indicating their widespread occurrence in the ambient environments.^11^, ^12^, ^62^ In parallel, detrimental effects such as reproductive and developmental toxicity to mammals, acute/chronic aquatic toxicity, and causing allergic contact dermatitis to occupational workers, of several broadly adopted PPDs have also been well recognized.^63^, ^64^, ^65^

**FIGURE 5:**
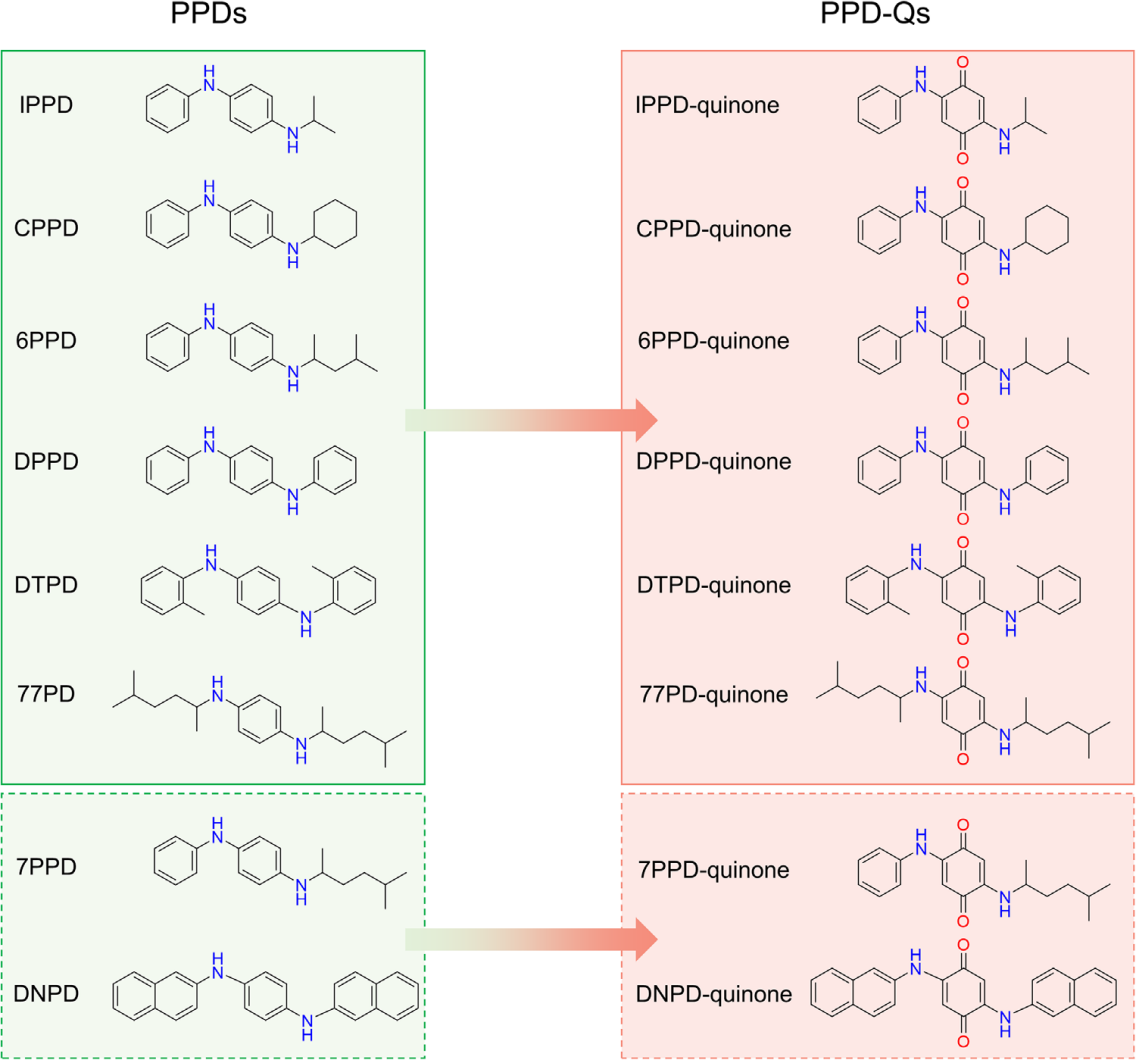
Transformation framework of *para*‐phenylenediamine antioxidants to their derived quinones. Chemicals in the solid line box represent identified transformations while those in dotted box represent possible transformations.

In 2020, a study published in *Science* first revealed that the quinone derivative of 6PPD (namely 6PPD‐quinone) could lead to the acute mortality (24‐h LC_50_ of 95 ng/L) of coho salmon (*oncorhynchus kisutch*) in the Northwest Pacific.^66^, ^67^ By applying multidimensional semipreparative HPLC coupled with qTOF MS in full scan/DDA modes, the toxicant 6PPD‐quinone was screened from more than two thousand tire rubber leachate molecules, which finally unraveled the mysteries of “urban runoff mortality syndrome,” that is, acute and widespread mortality of coho salmon in streams/rivers during rainfall events. Since then, research interests in the environmental occurrences and toxicities of *para*‐phenylenediamine quinones (PPD‐quinones) have greatly arisen. Huang et al. first identified the occurrence of 6PPD‐quinone in multiple dust samples collected from roads, parking lots, houses, and vehicles.^68^ The confirmation of 6PPD‐quinone is conducted with matched fragments of that reported by Tian et al.^66^ and the same retention time of all the packed ion transitions operated in MRM mode. Due to the deficiency of standard, the concentration of 6PPD‐quinone in the environmental compartments was semi‐quantified as 32.2‐80.9 ng/g based on the relative peak intensity of 6PPD‐quinone obtained from LC‐QqQ MS. Not far from that, the level of 6PPD‐quinone in PM_2.5_ was determined by UPLC equipped with a Xevo TQ‐XS QqQ MS (UHPLC‐MS/MS) in MRM mode, with the available commercial standard of 6PPD‐quinone.^11^ The median concentrations of 6PPD‐quinone among several megacities in China were estimated as 1.7‐6.7 pg/m^3^. Similarly, the level of 6PPD‐quinone in road dust collected in Tokyo, Japan was also investigated by utilizing an LC‐MS/MS system operated in MRM mode and the obtained concentration was normalized with the total organic carbon (OC) of the dust leachate.^69^ It is suggested that a potential generation of 6PPD‐quinone from traffic‐related sources with a higher level of 6PPD‐quinone was identified in samples collected from arterial roads with high traffic volumes (median: 8.6 µg/g‐OC) than residential roads with lower traffic volumes (median: 6.3 µg/g‐OC). Besides 6PPD‐quinone, a variety of other PPDs derived quinones including *N*‐isopropyl‐m'‐phenyl‐*p*‐phenylenediamine quinone (IPPD‐quinone), *N*‐phenyl‐*N*'‐cyclohexyl‐*p*‐phenylenediamine (CPPD‐quinone), *N,N*'‐diphenyl‐*p*‐phenylenediamine quinone (DPPD‐quinone) and *N,N*'‐bis(methylphenyl)‐1,4‐benzenediamine (DTPD‐quinone) were also identified in the PM_2.5_ samples collected in Hong Kong, with UPLC‐orbitrap MS suspect screening approach.^62^ By cross‐validation with synthesized standards, the detailed fragmentation patterns of such novel PPD‐quinones were clarified. Combined with the UPLC‐QqQ MS (MS/MS) platform using MRM mode, detailed quantification results regarding concentration and composition profiles of these emerging TPs among multiple environmental matrices, i.e., runoff water, roadside soil, and air particulates, were also specifically determined. One of the novel PPD‐quinone, IPPD‐quinone, was recently detected by LC‐QqQ TurboIonSpray ESI MS (LC‐MS/MS) in the electronic waste dust collected in South China with a median concentration of 363 ng/g.^70^ By referring to the fragmentation pathway information of PPD‐quinones, a new PPD‐quinone named *N,N*'‐bis(1,4‐dimethylpentyl)‐*p*‐phenylenediamine quinone (77PD‐quinone) has been recently identified using HRMS‐based suspect‐screening approach, in the PM_2.5_ samples collected from two megacities in China.^12^ The results revealed the comprehensive environmental characteristics including the potential sources, influencing factors, and spatiotemporal variations of these PPD‐quinones. Considering the similar usage and transformation pathways of PPDs, other frequently adopted PPDs may also undergo a potential transformation, leading to a wide variety of quinones (dotted box in Figure 5). Besides that, the enantiomers of 6PPD‐quinone also deserve enough concern as a recent study has revealed that *rac*‐6PPD‐quinone and *S*‐6PPD‐quinone were more hazardous than *R*‐6PPD‐quinone by 1.9 and 2.6 times, respectively, and the formation concentrations of *S*‐6PPD‐quinone and *R*‐6PPD‐quinone in 6PPD water solutions were greater than the LC_50_ values for *O. kisutch* and *O. mykiss*.^71^ Auto preparative LC system equipped with Superchiral R‐OJ and RIG columns were utilized to separate the enantiomers of 6PPD and 6PPD‐quinone, while their absolute configuration were confirmed by comparing the calculated the electronic circular dichroism spectra and measured the circular dichroism spectra. Then a supercritical fluid chromatography‐tandem mass spectrometer (SFC‐MS/MS) operated at MRM mode was employed to establish the enantioseparation procedure of 6PPD‐quinone.

Besides the identification and quantification, MS is also adopted in the health effects determination of PPD derivatives. Zhang et al. have determined 6PPD‐quinone pollution levels in eight size‐segregated particles (0.43‐10 µm) using UPLC QqQ MS/MS, and found an accumulative tendency of 6PPD‐quinone in coarse particles (9‐10 µm, 7.78‐23.2 pg/m^3^).^72^ Model simulations revealed different deposition fluxes of particle‐bound 6PPD‐quinone, which 89‐91% in workers’ head airways, 3.2‐3.8% in tracheobronchial, and 6.0‐6.9% in pulmonary alveoli areas of respiratory tracts, respectively. Another study by Wang et al. revealed the oxidative stress effects of five PPD‐quinones and found that DTPD‐quinone and 6PPD‐quinone were the most active species, with their oxidative potentials estimated to be 1.76 and 1.70 µM/(min·µM), respectively.^73^ By combining with their individual concentrations in PM_2.5_ accessed by UPLC‐ESI MS/MS, the contributions of PPD‐quinones to the total oxidative potential of PM_2.5_ were evaluated to be 2.9 ± 2.7% among the five investigated cities.

## 4 SUMMARY AND OUTLOOK

Atmospheric environment with the presence of ultraviolet light, ozone, free radicals, and various chemical components made it a giant reactor that promotes vast transformative reactions of emerging contaminants. The transformation products and pathways of such chemicals have been more and more clarified with the wide application of MS techniques. With the proceedings of computational chemistry and advanced MS technologies, an increasing number of studies have combined in‐house MS‐based experiments and in‐silicon simulation to attain high efficiency and high accuracy screening of unknown transformation products. Considering the specific chemical composition of PM_2.5_ is still constrained; additional research is still needed to fill the existing gap of a multi‐residue analytical methodology for the determination of emerging contaminants and their intermediate degradation products. It is expectable that transformation chemicals will gain more environmental concerns in the next future considering their ubiquitous distribution and potential toxicities. Correspondingly, the necessity for developing advanced MS‐based analytical platforms, including high sensitivity/resolution MS instruments, associated high performance separation systems, and relevant analytical strategies/frameworks is becoming increasingly apparent.

## AUTHOR CONTRIBUTIONS

Writing–original draft (lead), conceptualization (supporting): Wei Wang. Writing – original draft (supporting), writing – review and editing (equal): Guodong Cao. Software (lead), writing – review and editing (equal): Jing Zhang. Methodology (lead), writing – review and editing (equal): Han Qiao. Formal analysis (lead), writing – review and editing (equal): Fuyue Wang. Conceptualization (lead); Writing – original draft (supporting), writing – review and editing (equal): Zongwei Cai.

## CONFLICT OF INTEREST

The author declares that there is no conflict of interest.

## Data Availability

Data sharing not applicable to this article as no datasets were generated or analyzed during the current study.
